# Beyond Appearances: The Hidden Coat Diversity of the Sicilian Mastiff Revealed by Integrated Phenotypic and Genetic Analyses

**DOI:** 10.3390/vetsci13090958

**Published:** 2026-09-13

**Authors:** Arianna Bionda, Viviana Floridia, Paola Crepaldi, Luigi Liotta

**Affiliations:** 1 Department of Agricultural and Environmental Sciences–Production, Landscape, Agroenergy, University of Milan, 20133 Milan, Italy; arianna.bionda@unimi.it (A.B.); paola.crepaldi@unimi.it (P.C.); 2 Department of Veterinary Sciences, University of Messina, Viale Giovanni Palatucci, 98168 Messina, Italy

**Keywords:** dog, coat colour, genetics, breed standard, *ASIP*, *MC1R*, *KRT71*

## Abstract

Coat colour is one of the most recognizable and traditionally selected traits in dog breeds. However, despite its apparent simplicity, it represents a biologically complex phenotype, shaped by the interaction of multiple genetic loci, modifier genes, and variable phenotypic expression, challenging standardized phenotypic classification. This study presents the first characterization of coat colour and texture in the Sicilian Mastiff, integrating pedigree records, photographic assessment, and genetic analyses. Breeder-declared pedigree coat colour was examined for 760 dogs to evaluate terminology and its consistency, while photographs from 197 individuals enabled standardized phenotypic classification. Fifty dogs, selected within a monitored subset, were genotyped for 40 coat-related variants using the MyDogDNA™ commercial test, with additional analysis of *KRT71* curly-related variants in nine animals. Pedigree records included 115 terms for only 23 colour categories, revealing substantial inconsistency in terminology. Photographic evaluation identified considerably greater phenotypic diversity than registry data, showing that complex traits like brindling, ticking, melanistic masks, and white markings were frequently omitted or simplified; for example, only 8/64 Brindle black and tan dogs were identified as such in the registry. Genetic analyses confirmed segregation of alleles influencing eumelanin/pheomelanin distribution, dilution, white spotting, and hair texture—including both *KRT71* variants contributing to the characteristic curly coat of the breed—, while no known variants associated with merle, harlequin, cocoa, or albinism were detected. Our findings could provide valuable support to the breed association in requesting that the Italian Kennel Club (ENCI) revise the breed standard terminology and coat colour recording process, including allowing updates during growth, to improve clarity and consistency. Finally, requiring photographic documentation for registration applications could improve the reliability of coat colour assessment.

## Simple Summary

Coat colour is one of the most recognizable features of dog breeds, but describing it accurately can be difficult because several genetic factors can influence its appearance. This study provides the first detailed description of coat colour and hair texture in the Sicilian Mastiff. We examined coat colour information recorded in the pedigrees of 760 dogs, photographs of 197 dogs, and genetic results from 50 animals among a subset of monitored dogs. Pedigree records contained 115 different terms for only 23 colour categories, showing considerable inconsistency in the terminology used by breeders. Photographs revealed greater variation than pedigree records, as characteristics such as brindling, ticking, melanistic masks, and white markings were often omitted or simplified. Genetic testing confirmed the presence of variants affecting coat colour and hair texture, including two variants contributing to the characteristic curly coat of the breed. No known genetic variants associated with merle, harlequin, cocoa, or albinism were detected. These findings may help the breed association and the Italian Kennel Club improve coat colour terminology and recording procedures: for example, allowing records to be updated as dogs grow and requiring photographs for registration could make coat colour records more accurate and consistent.

## 1. Introduction

*Canis lupus familiaris* (Linnaeus, 1758) is one of the most beloved domestic animal species, with hundreds of breeds recognized worldwide, each distinguished by specific behavioural, functional, and morphological traits, typically described in their respective standards [1,2]. Among these, coat colour and texture have always been important traits in breed differentiation due to their high variability and distinctiveness, making them prime targets of human-driven selective pressures [3]. Consequently, the description and acceptance of coat colours and patterns in breed standards have also evolved over time, reflecting not only advances in breed characterization and historical knowledge but, in some cases, changing esthetic preferences or decisions made within the breeding community [4,5].

Although coat colour is often regarded as a classical example of simple Mendelian inheritance, its phenotypic expression and underlying genetic architecture are considerably more complex. Variation in coat colour and hair structure has been extensively studied to understand the genetic basis of these traits and their transmission across generations, revealing a complex architecture involving multiple interacting genetic factors that regulate pigment type, distribution, and patterning in hair follicles [4,6,7].

Coat colour in dogs is determined by the synthesis and distribution of two melanins produced by melanocytes: eumelanin (black/brown) and pheomelanin (red/yellow) [4,6,8]. Melanogenesis begins with the conversion of tyrosine into L-DOPA and subsequently DOPAquinone by tyrosinase, encoded by the *TYR* (tyrosinase) gene, within melanosomes [9]. DOPAquinone represents the branching point of the melanogenic pathway, from which pigment synthesis proceeds toward either eumelanin or pheomelanin depending on the activation state of *MC1R* (melanocortin 1 receptor). Activation of *MC1R* by α-melanocyte-stimulating hormone (α-MSH) upregulates melanogenic enzymes and favours eumelanin synthesis [10]. Conversely, the agouti signalling protein encoded by *ASIP* antagonizes *MC1R* signalling, reducing receptor activity and shifting pigment production toward pheomelanin [11,12]. In dogs, this regulatory pathway is further modified by *CBD103*, which encodes β-defensin 103. Unlike the canonical mammalian pathway, canine β-defensin 103 can competitively bind to MC1R, preventing ASIP-mediated inhibition and maintaining receptor activity, thereby promoting eumelanin production independently of *ASIP* expression [7,13,14,15,16,17]. Consequently, while spatial and temporal variation in *ASIP* expression contributes to pigment patterning, the interaction among *MC1R*, *ASIP*, and *CBD103* plays a central role in determining the final coat colour phenotype in dogs.

Pigment intensity and modification are further influenced by additional genes. *TYRP1* (tyrosinase-related protein 1) loss-of-function variants alter melanosome structure and eumelanin synthesis, thereby impairing the maturation of brown eumelanin precursors into black eumelanin [18]. *MLPH* (melanophilin) controls pigment dilution through impaired melanosome transport within melanocytes, reducing pigment intensity, with effects especially evident on eumelanin (e.g., black to blue, brown to lilac) [19,20]. Additional genes can modulate the intensity and shade of pigmentation, such as *MFSD12* (major facilitator superfamily domain containing 12) and *KITLG* (KIT ligand), which influence pheomelanin intensity and overall pigmentation saturation, respectively [21,22].

In addition to the control of eumelanin/pheomelanin switching and pigment intensity, coat colour variation also depends on the spatial distribution of pigment. White spotting and patterning are largely governed by *MITF* (melanocyte inducing transcription factor or microphthalmia-associated transcription factor), which is critical for melanocyte development, survival, and migration [8,23]. Within depigmented areas, additional variation can arise through variants in genes such as *USH2A* (usherin), which produce the roan pattern, characterized by interspersed pigmented hairs [24,25]. More extreme depigmentation patterns, such as merle, are associated with *PMEL* (premelanosome protein), where a retrotransposon insertion disrupts eumelanin deposition, producing patchy dilution and can have pleiotropic effects on eye development and hearing [26,27]. *PSMB7* (proteasome 20S subunit beta 7) acts as a modifier of the merle pattern, further enhancing depigmentation in diluted regions and producing the so-called harlequin phenotype [28]. Complete loss of pigmentation (albinism) can result from defects in genes involved in melanin synthesis and melanosome function, including *TYR*, *SLC45A2* (solute carrier family 45 member 2), and *OCA2* (OCA2 melanosomal transmembrane protein), which impair tyrosinase activity and melanosomal homeostasis, thereby preventing effective melanin production [29,30,31].

Not only is coat colour a determinant of breed identity, but from a zootechnical perspective, hair texture is also an important component of breed-specific morphology and may contribute to breed characterization and selection, while influencing the functional properties of the hair shaft and the growth dynamics of the hair follicle. Key genes include *RSPO2* (R-spondin 2), which affects hair growth and the presence of furnishings by modulating Wnt signalling pathways [6,32]; *FGF5* (fibroblast growth factor 5), a regulator of the hair growth cycle where loss-of-function variants result in prolonged anagen and thus longer hair [6,33]; and *KRT71* (keratin 71). *KRT71* encodes a type II intermediate filament protein that is highly expressed in the inner root sheath and hair shaft, where it contributes to the structural organization and mechanical properties of the hair fibre. Consequently, variation in *KRT71* can affect hair shape and texture. At present, two functional *KRT71* variants, commonly referred to as *C1* and *C2*, have been associated with curly or wavy coat phenotypes in dogs. The presence of these variants has been observed across breeds belonging to multiple clades and functional groups, with both variants also observed within the same breed [6,34,35].

The combination of the genotypes across these loci, along with others that remain incompletely characterized, contributes to determining the visible phenotype of the animal. To date, breed standards are primarily based on traits that can be visually assessed by breeders, including the description of the accepted coat colours and type [36]. However, the phenotype does not always fully reflect the underlying genetic profile. Indeed, some alleles may be present without being externally manifested, leading to apparent discrepancies between genotype and observable phenotype. As a consequence, individuals may produce offspring with unexpected phenotypes due to hidden genetic variation [27,37,38]. In this context, knowledge of the genotype is of fundamental importance not only for the accurate selection of animals with desired characteristics, but also for achieving a more precise and objective description of colour and other heritable traits.

Indeed, in the Italian Kennel Club (*Ente Nazionale della Cinofilia Italiana*, ENCI) there is currently no rigorous and standardized system for the classification and recording of coat colours in official documents. Terminology often varies across breeds and, in some cases, even within the same breed, despite referring to similar genetic bases. Moreover, breeders are not always fully aware of the genetic mechanisms underlying coat colour and may, in some cases, rely on purely descriptive terms based on the animal’s appearance rather than standardized terminology referring to specific colours or patterns. This inconsistency highlights the need for more uniform and standardized criteria for defining coat colours, capable of reducing ambiguity and improving consistency between phenotype and genotype.

This need is particularly relevant for newly recognized breeds, where standardized phenotypic terminology can be adopted before inconsistent descriptions become established. One such breed is the Sicilian Mastiff (or Mannara dog), a native Sicilian livestock guardian dog recently recognized by the ENCI and characterized by a distinctive curly coat and multiple coat colours and patterns [39]. The aim of the present study was therefore to provide the first comprehensive characterization of coat colour and coat texture diversity across the entire registered population of the Sicilian Mastiff and provide the first data about their underlying genetic basis in a subset of individuals. By integrating pedigree records, standardized phenotypic assessment, and molecular genetic analyses, we further assessed the extent to which coat descriptions recorded in the ENCI registry reflect the breed’s phenotypic and genetic diversity.

## 2. Materials and Methods

### 2.1. Context of the Study

The Sicilian Mastiff is an ancient livestock guardian breed native to Sicily, historically selected to protect sheep and goats from predators within a traditional enclosure known as *mannara*, from which its alternative name, Mannara dog, derives. Archeological and historical evidence suggests that mastiff-type dogs have been present on the island since the Bronze Age, with possible later influences from Molossian and North African shepherd dogs [39]. Despite its long history and established presence in Sicily, efforts toward its formal recognition began only recently.

The recovery of the Sicilian Mastiff began in 2014 with the first breed gathering aimed at registering the founding dogs in the Open additional register (RSA, *Registro Supplementare Aperta*), the open studbook established for breeds undergoing formal recognition. Over the following years, the growing number of dogs enrolled in the RSA, together with genetic studies confirming the breed’s distinct ancestry, supported its case for official recognition [39]. In 2023, ENCI formally recognized the Sicilian Mastiff as an autochthonous breed and opened the Registry of recognized dogs (RSR, *Registro Supplementare Riconosciuti*), the closed studbook reserved for recognized breeds. Since then, with a view to future international recognition by the *Fédération Cynologique Internationale* (FCI), the recovery project has pursued two main objectives: the systematic screening of hereditary disorders (orthopedic, cardiac and ocular) within the breeding population, and the genetic characterization of coat colour variation to inform the definition of the new breed standard according to FCI nomenclature and format.

The Sicilian Mastiff breed standard [40], published in 2022, provides the following specifications regarding coat colour and texture:

- Coat: The outer coat is medium-long, with large, loose curls or pronounced waves; it is harsh to the touch, dense and close-lying. An undercoat is present, and the coat must not allow the skin to show through. The hair is short on the skull and muzzle.
- Colour: Black, fawn (ranging from cream to mahogany), light liver (“flea” colour), grey and brindle, all of which may vary in purity of shade. Any of the above colours may include white markings of varying extent.
  When white is present, the distribution known as the “Monaca” (Nun) pattern is preferred: white markings on the muzzle, including a blaze, as well as on the throat, chest, neck, feet and tip of the tail. Symmetry of the markings is not required. White must never be the predominant colour.
  All the above colours may display faint tan markings, which may also be brindled, above the eyes (the so-called “four-eyed” pattern), and on the muzzle, cheeks, chest and limbs. A characteristic feature of the breed is that these tan markings lighten and expand with age, eventually becoming a very pale ivory colour.
  Grey and brindle coats, whether solid or with white markings, are permitted but not desirable.
- Severe faults: Coat in which white is predominant; Coat with insufficient waviness or with curls that are too small and tight.
- Disqualifying faults: Solid white coat or any colour not specified in the standard; Straight, short or long coat that does not conform to the above description; Long, abundant feathering on the ears or limbs; Clipped or trimmed coat.

### 2.2. Pedigree, Identification Details, and Photographs

Data were collected from ENCI registries for the entire Sicilian Mastiff population, including dogs registered between October 2014 and April 2026, for a total of 760 records (data provided by ENCI). The available information included identification details, pedigree, dates of birth and registration, sex, and coat colour (as reported by the owner). The latter variable was analyzed to reconcile the different terminologies into a standardized nomenclature. Within the population officially registered in the ENCI Genealogical Books, 431 dogs are registered in the RSA (Open additional register), 305 to RSR (Registry of recognized dogs), and 24 to ROI (Register of Italian origin), with a total of 151 founders. Among ROI and RSR-registered dogs, approximately 72 regularly take part in official ENCI events, such as dog shows. These events are zootechnical evaluations in which participating dogs are assessed by accredited ENCI judges; as such, they constitute the most qualified and closely monitored breeding pool currently available for the breed. In fact, these 72 dogs have already undergone standardized health screening protocols, in line with the guidelines of ENCI and the FCI, including evaluations for hip and elbow dysplasia; official ophthalmologic examinations conducted according to the European College of Veterinary Ophthalmologists (ECVO) protocol; cardiological assessments performed through echocardiography; and behavioural evaluation through the ENCI Reliability and Balance Control test.

In addition to the aforementioned data, Samannara, the Sicilian Mastiff breed club, invited the owners of all registered dogs to submit photographs of their animals. The submitted photographs were screened, and images of puppies, images in which coat colour could not be reliably assessed, and images of dogs that could not be identified in the pedigree database were excluded. After screening, photographs of 197 dogs were retained, with multiple photographs available for some individuals. The representativeness of this subset relative to the registered population was assessed with respect to sex, year of birth, registered coat colour, breeder, and founder status (i.e., whether the dog was a founder, defined as having no registered ancestors, or a non-founder) using Fisher’s exact test, with *p*-values estimated by Monte Carlo simulation using 100,000 replicates when exact computation was computationally infeasible, as implemented in the fisher.test function of the R stats v. 4.4.1 package. All photographs were assessed by the same author, who classified each dog’s coat colour while blinded to the colour reported by the breeder or owner and recorded in the pedigree database. Although photographic assessment may be affected by lighting conditions or image quality, photographs for which coat colour was considered uncertain were excluded to minimize potential misclassification. However, the evaluation of all photographs by a single assessor, which precluded the assessment of inter-observer reliability, represents a limitation of the study. Concordance between pedigree- and photograph-based coat colour classifications was also assessed using Cohen’s K in JMP Student Edition 19.0.3 (JMP Statistical Discovery LLC, Cary, NC, USA).

### 2.3. Genetic Tests for Coat-Related Variants

For genomic analysis, we used data from a subset of 50 dogs selected from this group of 72 monitored individuals mentioned in the previous section: Samannara offered free health screening and genetic testing for all these dogs, and owners were free to decide whether to participate; although this sampling strategy may introduce some bias, these dogs represent the segment of the population most frequently evaluated and most likely to contribute to the next generation. Additionally, the representativeness of the subset of tested dogs within the entire registered population was assessed as described above for the photographic dataset.

Buccal swab samples for these dogs had previously been collected and submitted to the MyDogDNA™ genetic testing panel within the framework of official zootechnical evaluations carried out by Vetogene ENCI Servizi and the University of Messina; therefore, in the present study, we analyzed the existing genetic test results generated independently of this research. The panel screens approximately 270 variants associated with known hereditary diseases and about 50 variants linked to morphological traits. Of these, 40 variants at 20 different loci are specifically related to coat colour and texture. For all sampled dogs, owners also provided photographs, which enabled the assignment of coat colour according to a standardized nomenclature.

Additionally, as the Sicilian Mastiff typically presents a coat with large, open curls or pronounced waves [40] and the MyDogDNA™ panel tests for only one of the two known variants in the *KRT71* gene associated with curly hair (namely, *C1*), a subset of nine samples (selected among those with zero or one copy of *C1*) was further analyzed to assess the presence of both the *C1* and *C2* variants through “Curly (curled hair: C1, C2)” test performed by Laboklin GmbH & Co. KG (Bad Kissingen, Germany) (https://laboklin.com/it/esami/genetica/mantello-colore-struttura-lunghezza/dog/curly-curled-hair/, accessed on 27 August 2026).

## 3. Results

### 3.1. Pedigree-Recorded Coat Colours

A high degree of heterogeneity was observed among the 760 coat colour records, with a total of 115 different registered terms.

**Figure 1:**
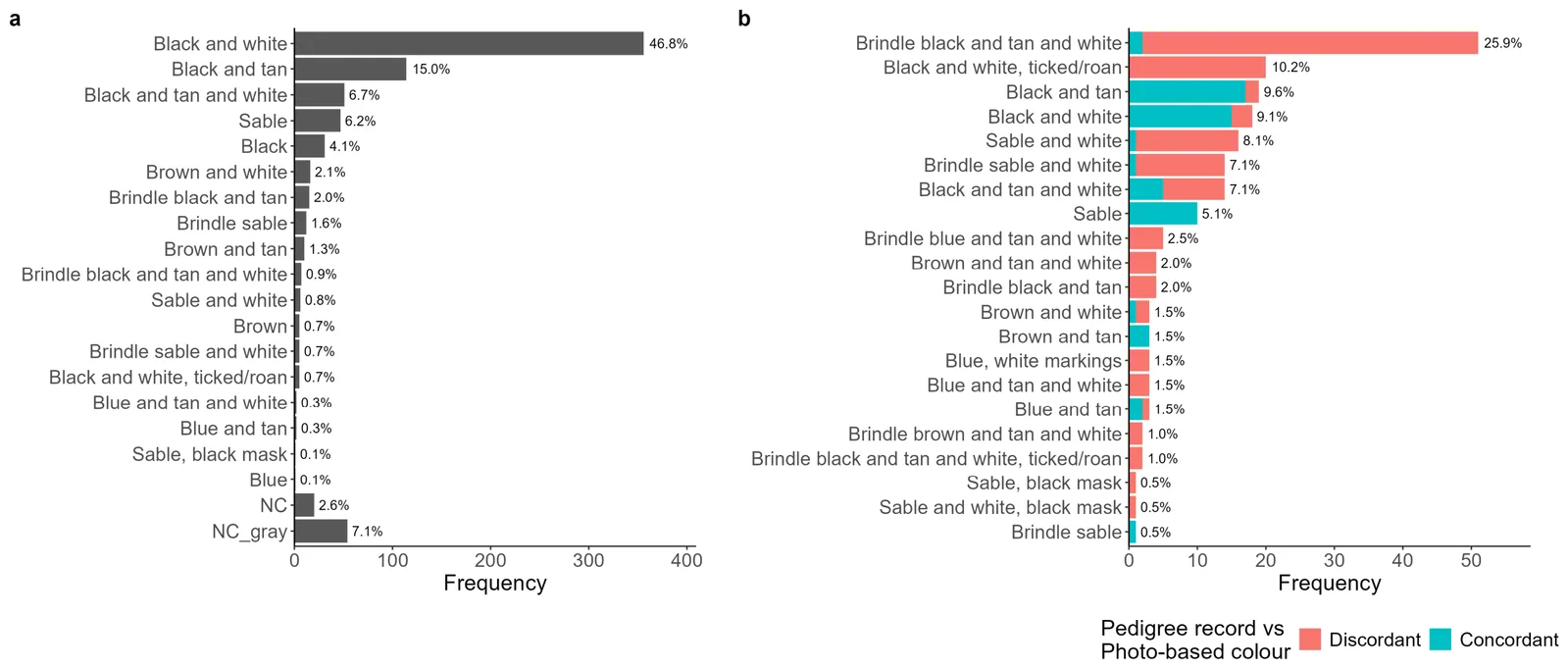
Distribution of coat colours in Sicilian Mastiffs based on pedigree-recorded colour descriptions ((**a**), n = 760) and photograph-based colour classifications ((**b**), n = 197). For clarity, dogs with extensive white patches and those with limited white markings were grouped into a single category. Roan and ticking were considered together. Black masks were distinguished only in sable dogs, as they are more readily identifiable in this coat colour. See Supplementary Tables S1 and S3 for more details. “NC” category includes terminology that could not be unambiguously interpreted as standardized coat colours, whereas “NC_gray” comprises entries containing the term “gray”.

A subset of descriptors, specifically 20 terms consisted of uncommon or subjective terminology that could not be unambiguously interpreted as standardized coat colours, such as “Brindle silver”, “Roan with white”, “Dove-gray”, “Charcoal gray”, and “Blue-black”. Additional 55 entries (7%) contained the term “gray,” either alone or in combination with other colour (e.g., “White and gray”, “Black and gray”), representing a particularly problematic category due to its broad phenotypic interpretation, which may encompass, for example, blue, roan, brindle sable, and brindle tan points. The remaining 685 entries were traced back to 23 different colours (Figure 1a, Supplementary Table S1).

The most frequently recorded coat was Black and white (350 individuals, 46%), followed by Black and tan without (115 individuals, 15%) or with white (50 individuals, 7%). The other colours have a very low or marginal frequency. For example, 6% (47 dogs) were reported as Sable without white markings, while 6 individuals were described as Sable and white, often with an indication, though not consistently reported, of pheomelanin intensity. 17 individuals were indicated as Brindle sable, with or without white; however, an indication of the eumelanin colour (e.g., black, blue, or brown) was never provided. Blue and Brown were reported, with different patterns, in 5 (0.7%) and 31 (4%) dogs, respectively, while no Lilac or Isabella were indicated; however, it should be noted that several blue-coated dogs were likely described as gray by their owners. Additionally, roan/ticking was indicated in five dogs only, and the presence of a melanistic mask in a single sable dog.

Among synonymous names, some differed in word order; this may, in certain cases, reflect variations in perceived colour distribution, although the distinction is not applied consistently (see results below). For example, the 356 Black and white dogs (including both those with white patches and those with white markings only), included “Black white” (n = 216), “White black” (n = 68) and “Black and white” (n = 32), in addition to specific indications of the location of small patches (e.g., “Black with white feet”, “Black with white marking on the chest”) or other similar variants.

**Figure 2:**
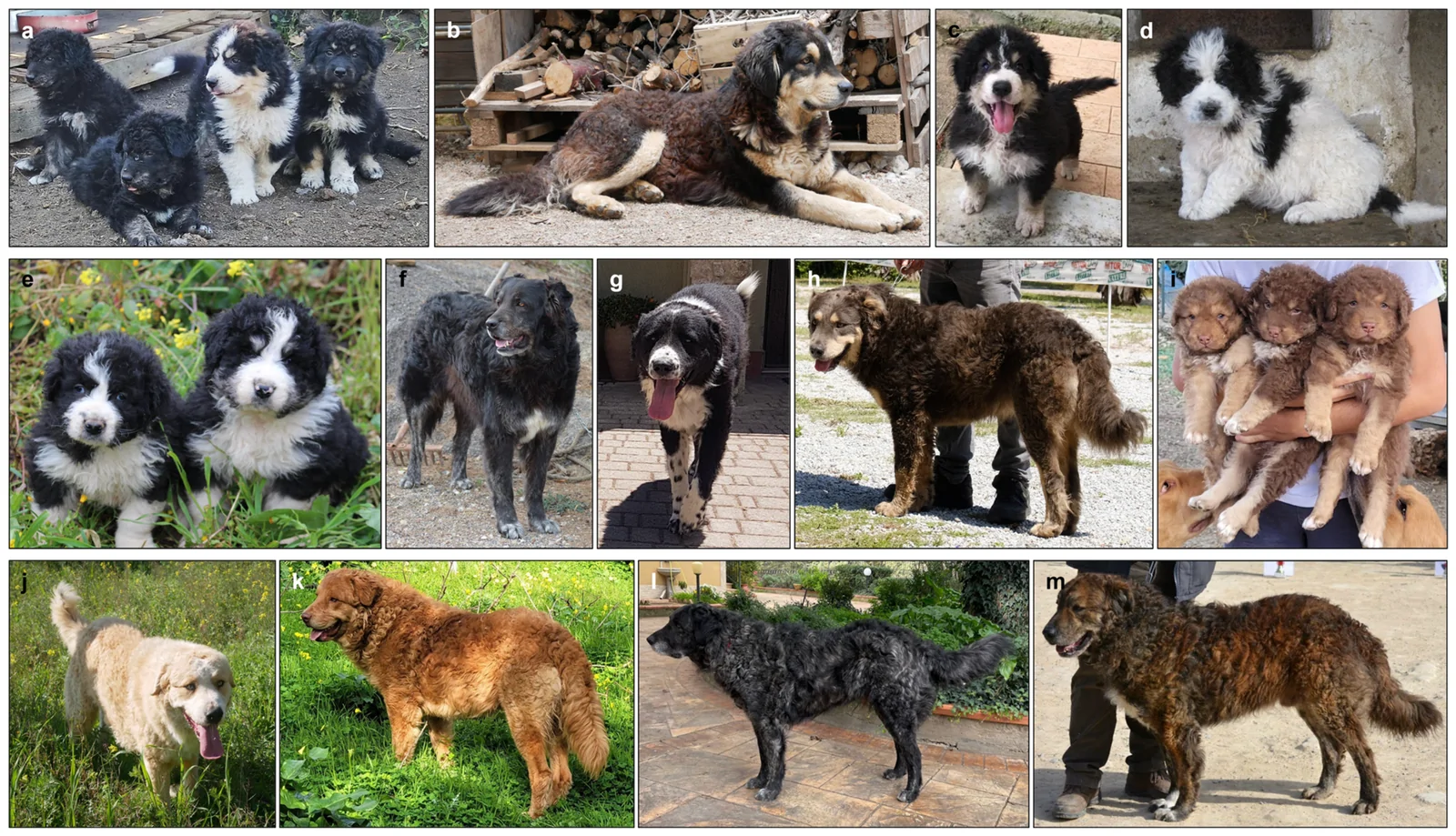
Main coat colours of the Sicilian Mastiff. (**a**) Puppies showing different coat colours: the two subjects on the left are Brindle black and tan with black mask and white markings (on feet and chest), with brindled tan points particularly visible on limbs and muzzle; the central subject is Black and tan and white, with a typical Irish-like pattern and the tan points identifiable on eyebrows; the subject on the right is Black and tan with black mask and white markings (on feet and chest). (**b**) Black and tan, with the typical tan points on muzzle, eyebrows, chest, and limbs. (**c**) Black and tan and white (commonly called Tricolour). The white patches follow an Irish-like pattern, while the tan points are identifiable above the eyes, on the cheeks, and on the distal and inner portions of the limbs. In most Sicilian Mastiffs, the tan markings are very pale, as also specified in the breed standard, and may therefore be difficult to distinguish from adjacent white patches. (**d**) Black and white puppy displaying a piebald pattern. The coat is predominantly white, with coloured areas typically occurring on the head (often together with a white muzzle and forehead blaze) and at the base of the tail, as well as patches of variable size and shape on the back. When white covers much of the muzzle, it may be difficult to determine whether tan points are also present. A predominantly white coat is considered a fault under the breed standard. (**e**) One puppy displaying a Black and white coat with the “Monaca” (“Nun”, similar to Irish) pattern, corresponding to the Irish spotting pattern (left), and one Black and tan and white puppy (right). Notice that the puppy on the left does not display tan eyebrows, whereas the one on the right does. (**f**) Brindle black and tan with white markings. The brindled tan points are especially visible on limbs and muzzle. (**g**) Ticked black and white, with the “Monaca” (“Nun”, similar to Irish) pattern. Ticking is clearly visible on limbs and muzzle. (**h**) Blue and tan, with the typical tan points on muzzle, eyebrows, chest, and limbs. (**i**) Brown and tan with white markings on feet and chest. (**j**) Shaded sable and white, with low-intensity pheomelanin. (**k**) Sable with intense pheomelanin. Note also the brown nose, which likely indicates homozygosity for the *b* allele at the B locus. (**l**) Brindle sable with white markings and a black mask. In this individual, eumelanin expression predominates. (**m**) Brindle sable with white markings. Compared with the dog shown in panel (**l**), pheomelanin expression is more evident and intense.

The designation “Monaca” (meaning “Nun”, Figure 2) was explicitly reported in 21 cases (3%). This pattern, which is also mentioned in the ENCI breed standard, resembles the typical Irish pattern, it being defined by the presence of a white blaze on the muzzle and white patches involving throat, chest, neck, socks and tip of the tail. However, such pattern-level detail was inconsistently recorded across the dataset, with most entries limited to basic colour descriptions. In addition, complex patterns, such as brindle combined with black and tan, were rarely identified explicitly, representing only 2% (n = 15) of entries.

Despite the inconsistencies, all recorded coat colours appeared broadly compatible with breed standards, and no clearly out-of-standard individuals were identified based solely on the reported colour descriptors.

### 3.2. Comparison Between Photo-Based and Recorded Colour

The representativeness of the dogs with available photographs (n = 197) relative to the whole registered population (n = 760) was assessed using Fisher’s exact test. No significant differences in the distributions of the two datasets were observed for Breeder name (*p* = 0.57), Sex (*p* = 0.34), Year of birth (*p* = 0.93; range: 2009–2025), Standardized pedigree-based colour (*p* = 0.19), or Founder status (*p* = 0.49; photographs were available for 44 founders).

Photographic assessment of 197 registered dogs identified 48 distinct coat-colour phenotypes, whereas pedigree records recognized only 19 standard coat-colour categories, highlighting a substantially greater level of phenotypic variation (Supplementary Table S2). Moreover, the coat-colour distribution derived from photographs differed markedly from that based on the recorded pedigree colours (Figure 1b, Supplementary Table S3). For example, although Black and white was by far the most frequently recorded colour, it was identified in only 18 dogs (9%) based on photographic assessment, including four dogs displaying only white markings. Treating the standardized pedigree-based descriptor “white markings” as equivalent to the photograph-based descriptor “and white”, and vice versa, pedigree- and photograph-based coat colour classifications were concordant for 60 dogs (30%; Cohen’s K ± SE: 0.27 ± 0.031). A further 20 individuals showed only minor inconsistencies, such as the omission of a mask or white markings, resulting in an overall concordance of 41% (Cohen’s K ± SE: 0.37 ± 0.035). Note that all individuals whose owner-recorded colour could not be assigned to a specific category (coded as NC or NC_gray) were considered discordant. The most frequent concordant classifications were Black and tan (n = 17), Black and white (n = 12), and Sable (n = 10).

Brindled tan points were common, being observed in 64 dogs (32%), but this pattern was explicitly recorded in only 8 cases (13%). Specifically, in 44 dogs (69%), only the base colour (e.g., Black and white) was recorded, with no indication of either tan points or brindling, whereas in 6 dogs (9%) tan points were reported but brindling was omitted.

Non-brindled tan points were also well represented, occurring in 46 dogs (23%), and were generally recorded as such. However, in 7 dogs (15%), only the base colour was reported, with no mention of tan points.

A total of 15 dogs (8%) were identified as Brindle sable, but this pattern was recorded in only five cases (33%). The remaining dogs were registered as Black (n = 5), Brown (n = 3), Fawn (n = 1), or Black and gray (n = 1).

Among the 28 (14%) clear or shaded Sable dogs, six (21%) exhibited intense pheomelanin, all coming from the same family. Notably, all sable dogs were recognized as such by their owners, although a variety of colour terms were used, including cream, fawn, and honey.

With regard to dilution, 128 dogs were black-based, 14 blue-based, and 12 brown-based. However, the base colour was discordant with the photograph-based classification in six cases: three blue dogs were registered as black, while two brown dogs were recorded as black and red, respectively, in addition to those described using gray or other non-standard terms. Indeed, the term “Blue” was reported for only one dog.

Most dogs displayed white patches. Sixty dogs (30%) had limited white markings, such as white feet and/or a white chest, but these markings were not reported in 33 cases (55%). 94 dogs (48%) exhibited more extensive white patches, predominantly corresponding to the Irish spotting pattern; nevertheless, the presence of white was omitted in 15 cases (16%).

A melanistic mask could be identified in 19 (10%) photographs, but it was never reported by owners. Similarly, 22 individuals (11%) displayed ticking or roaning, yet these patterns were never indicated in the registered colour descriptions. Among the non-standard colour terms, we found a Sable with black mask dog described as “Sooty fawn” (“*Fulvo carbonato*” in Italian), a Brindle black and tan dog with white markings described as “Black and beige”, and a Brindle sable with white markings described as “Brindled brown”. In addition, 11 colour descriptions (6%) included the term “gray”: most of these (n = 8) referred to dogs with a blue base colour, whereas the remaining cases described a Brown and tan dog, a (shaded) Sable dog, and a Brindle sable dog.

We also examined whether the order of the colour terms reported in the pedigree was associated with the extent of white patterning. Among dogs with available photographs, we compared records in which the base colour preceded “white” (e.g., Black and white) with those in which “white” preceded the base colour (e.g., White black); dogs whose pedigree-based colour specified the location of the white markings were excluded. The BaseColour–White category comprised 69 dogs (Black white, Black and white, Gray with black, and Gray and white), of which 20 (29%) had limited white markings, 48 (70%) displayed an Irish-like pattern (an example of this pattern is reported in Figure 2e,g), and one was piebald (an example of this pattern is reported in Figure 2d). Notably, the latter dog was also roan, causing the white areas to appear gray. The White–BaseColour category comprised 18 dogs (White and black, White black, White gray, White cream, and White black nun), of which six (33%) were piebald, 11 (61%) displayed an Irish-like pattern, and one had limited white markings. Thus, the base colour generally preceded the term “white” in records of dogs with limited white markings, whereas the reverse order was more common in piebald dogs; instead, dogs with an intermediate extent of white, such as an Irish-like pattern, were recorded using either order.

With regard to out-of-standard traits, we identified ten dogs with predominantly white coats (Piebald spotting), a severe fault according to the breed standard, and two dogs with a clearly smooth coat texture, which is considered a disqualifying fault.

### 3.3. Genetic Tests for Coat-Related Variants

The distribution of the tested dogs (n = 50) was compared with that of the whole registered population (n = 760) using Fisher’s exact test. No significant differences were found between the two groups for Breeder name (*p* = 0.1258), Sex (*p* = 0.47), Year of birth (*p* = 0.09), Standardized pedigree-based colour (*p* = 0.45), or Founder status (*p* = 0.28).

The genetic architecture underlying coat colour in the studied population was first examined in relation to the eumelanin–pheomelanin switch mechanism, primarily governed by variation and interaction at the E, K, and A loci. At the E locus, 18% of the tested dogs carried the *e*^1^ allele and all were heterozygous. Consequently, pheomelanic individuals were attributable to the *k^y^k^y^ A^y^-* genotype (dominant yellow/sable; n = 3) rather than recessive red. Nevertheless, the relatively high frequency of *e*^1^ suggests that recessive red phenotypes may occur within the broader population. All eumelanic coats were associated with the presence of the *K^B^* allele, as no *a* allele at the A locus was detected.

**Figure 3:**
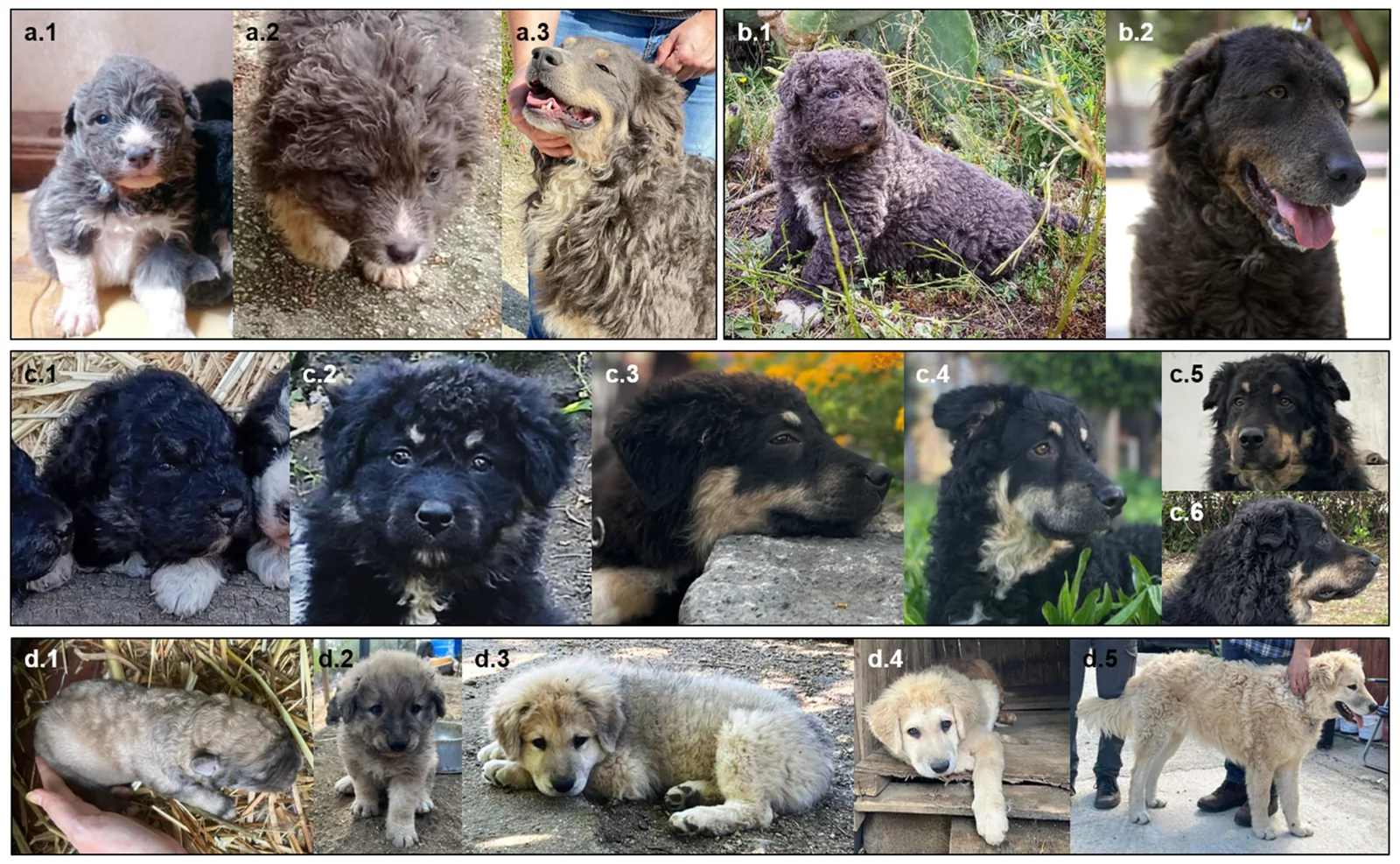
Coat colour changes with age. (**a**–**c**) Three dogs carrying the *E^m^* allele at the E locus, each showing a distinct melanistic mask early in life that progressively faded with age. (**a**) Blue and tan with white markings (on the feet, chest and nose) at 20 days (**a.1**), 50 days (**a.2**), and 3 years of age (**a.3**). The melanistic mask, which covers the tan markings at a young age, is almost completely absent in the adult (**a.3**), where tan markings on the muzzle and eyebrows become clearly visible. (**b**) Brindle blue and tan with white markings (on the feet and chest). As a puppy ((**b.1**), 15 days), the muzzle appears entirely blue due to the melanistic mask, whereas brindled tan markings become clearly visible, especially on the muzzle, in adulthood ((**b.2**), 10 months). The highly curly puppy coat also obscures the brindled pattern on the limbs during early life. (**c**) Black and tan with white markings dog at 15 days (**c.1**), 35 days (**c.2**) 12 weeks (**c.3**), 1.5 years (**c.4**), and 4 years of age (**c.5**,**c.6**). Tan points on the muzzle are barely visible in the first two images (**c.1**,**c.2**) but become progressively more evident with age. In the adult (**c.5**,**c.6**), only light black shading remains on the rostral muzzle, together with a black chin. (**d**) Shaded sable and white dog at 7 days (**d.1**), 45 days (**d.2**), 70 days (**d.3**), 4 months (**d.4**), and 18 months of age (**d.5**). This individual had a markedly darker coat as a puppy, while eumelanic shading became minimal in adulthood. Black hair on the muzzle can mimic a melanistic mask during early development (**d.1**,**d.2**), but this effect is independent of the E locus and disappears very early during growth.

Almost half of the dogs carried at least one copy of the *E^m^* allele, which is typically associated with a melanistic mask. Despite this, the mask was rarely recorded in phenotypic descriptions. This likely reflects both the difficulty of visually identifying the mask in certain coat types (e.g., dark Brindle sable or (brindle) Black and tan patterns) and its complete masking in solid black individuals. Three dogs heterozygous for *E^m^* did not exhibit a clearly visible mask, representing the only observed genotype–phenotype discrepancy at this locus. Notably, puppyhood photographs revealed the presence of a melanistic mask that had faded by adulthood, remaining only as subtle residual pigmentation in some individuals and becoming no longer clearly discernible in others (Figure 3a–c). The only additional variants identified at the E locus were *e^A^* (n = 3) and *e^H^* (n = 1).

At the K locus, 74% of dogs carried at least one copy of either *K^B^* or *k^br^*. However, the lack of distinction between these alleles limited detailed phenotypic-genetic concordance evaluation. This is particularly relevant given the known difficulty in distinguishing Brindle black and tan (*k^br^k^br^* or *k^br^k^y^*) from solid Black (*K^B^_-_*) phenotypes.

Analysis of the A locus showed that the *a^t^a^t^* genotype was the most common (74%), corresponding to tan points patterning when expressed. The *A^y^a^t^* genotype, associated with Sable colouration, was observed in 26% of dogs. The *a^w^* and *a* alleles were not identified among the tested dogs.

The variant of the *RALY* gene thought to be associated with saddle tan had a frequency of 28%, although only two dogs were homozygous. Neither of these individuals exhibited a saddle pattern; instead, both displayed Black/Blue and tan coats.

Regarding dilution, at the D locus (*MLPH*) the *d*^1^ allele, responsible for dilution of black to blue and chocolate to Isabella, had a frequency of 38%. Four dogs were homozygous and expressed a blue coat. No additional recessive dilution variants were identified at this locus. At the B locus (*TYRP1*), two recessive variants were detected: *b^c^* (1%) and *b^s^* (21%). Only two dogs were homozygous (*b^s^b^s^*) and expressed a brown coat. No Isabella phenotypes were observed, despite the presence of both *b* and *d* alleles in the population. Notably, this colouration is not included in the breed standard.

Variation at the I locus (*MFSD12*) supported phenotypic observations of generally light tan markings in Black and tan dogs, as also described in the breed standard. While cream or honey phenotypes were most common, more intensely pigmented pheomelanic individuals were also observed. The high frequency of the *i* allele (88%) and absence of the *II* genotype further support a general trend toward reduced pheomelanin intensity.

At the S locus, only the *s^p^* allele was tested. White spotting is now understood to result from regulatory variation at the *MITF* locus together with additional modifier genes. Therefore, the same genotype may be associated with different white spotting phenotypes, particularly across different breeds [23]. No homozygous *s^p^s^p^* Sicilian Mastiffs were identified, consistent with the apparent absence of predominantly white dogs in the sampled population. Approximately one-third of individuals were heterozygous, most of whom displayed an Irish spotting pattern, while a smaller number exhibited only minimal white markings (e.g., toes or chest). Among dogs lacking the *s^p^* allele, variation in white patterning was still observed, including Irish-like spotting, limited markings, or complete absence of white.

Ticking/roan patterning is not explicitly described in the breed standard, but at the same time is not considered a fault during show evaluations, and was observed among the analyzed dogs. Four dogs were heterozygous for the *T^r^* allele at the *T* locus, three of which showed visible roaning in white areas, whereas the fourth had only minimal white markings. Additionally, five dogs lacking the *T^r^* allele exhibited ticking on the limbs, supporting previous evidence that ticking and roan are regulated by distinct genetic variants [24]. It should be noted, however, that phenotypic discrimination between roan and heavy ticking may be unreliable in dogs with long, curly coats, particularly when white markings are limited.

No variants associated with merle, harlequin, cocoa, or albinism were detected among tested dogs.

With respect to coat texture, analysis of the *KRT71* gene revealed that 31 dogs did not carry *C1*, while 18 were heterozygous based on initial testing; however, all individuals displayed clearly curly or at least wavy coats. Subsequent testing for the *C2* variant in a subset of nine dogs identified at least one dominant curl-associated allele in all cases (three *C1C2*, two *C2C2*, one *C1c*, and three *C2c*, assuming the alternative allele is *c*). The presence of heterozygous individuals suggests that smooth-coated offspring could potentially occur, unless additional, uncharacterized variants contribute to coat curliness in this population.

Concerning *FGF5* variants, almost all dogs were homozygous for long hair-related alleles (46 *lh*^1^*lh*^1^ and one *lh*^1^*lh*^3^), with only two individuals carrying a single *lh*^1^ allele. These two dogs did not show clear phenotypic differences in coat length compared to the rest of the sample.

Variation in the *MC5R* gene, associated with shedding, showed that 10% of dogs were heterozygous for the *sd* allele and one dog was homozygous. This variant is typically linked to reduced shedding and may also influence hair length.

Finally, no variants were identified in genes associated with furnishings (*RSPO2*), alopecia (*SGK3*), ridge formation, or hairlessness (*FOXI3*).

Individual results for all tested dogs are reported in Supplementary Table S4, whereas the frequencies of dogs heterozygous or homozygous for each tested allele are summarized in Table 1.

Additionally, among the 50 genetically tested dogs, 21 parent–offspring pairs and two trios comprising a dam, sire, and offspring were identified. No Mendelian incompatibilities were detected at the tested coat-related variants in any of these groups.

## 4. Discussion

Coat colour and coat texture are among the most visible breed-defining traits and are often subject to conscious or unconscious selection by breeders. Beyond their esthetic importance, pigmentation traits can provide information on breed history, population structure, and ancestry [37,41,42,43,44,45], and in some cases have been associated with behavioural tendencies [46,47,48,49] or health-related conditions [4,50,51]. Accurate characterization of coat colour variation is therefore relevant not only for breed standardization and conservation, but also for understanding the genetic diversity maintained within the population. Within this context, the present study provides the first integrated description of coat colour and hair texture variation in the Sicilian Mastiff (also called Mannara dog), combining pedigree records, photographic evaluation, and molecular genetic analyses.

A remarkable heterogeneity was observed in the terminology used to describe coat colour. More than one hundred different descriptors were identified, including numerous synonymous expressions, variations in word order, and subjective terms lacking a clear genetic or phenotypic definition. Part of this diversity likely reflects genuine variation in phenotype. For example, Sable dogs were described using terms such as cream, honey, fawn, and sable, which may partly correspond to differences in pheomelanin intensity, although these designations were not applied consistently. Likewise, some descriptors appeared to reflect the extent or anatomical distribution of white markings rather than distinct coat colour categories. In contrast, approximately 20 colour terms represented uncommon or highly subjective descriptions that could not be unambiguously assigned to standardized phenotypic classes.

However, the most important finding was not the diversity of terminology itself, but the discrepancy between registered and observed phenotypes. Comparison with photographic records demonstrated that pedigree descriptions frequently simplified complex colour patterns into a limited number of broad categories. Consequently, registry data substantially underestimated the true diversity of coat colour variation present within the breed.

**Figure 4:**
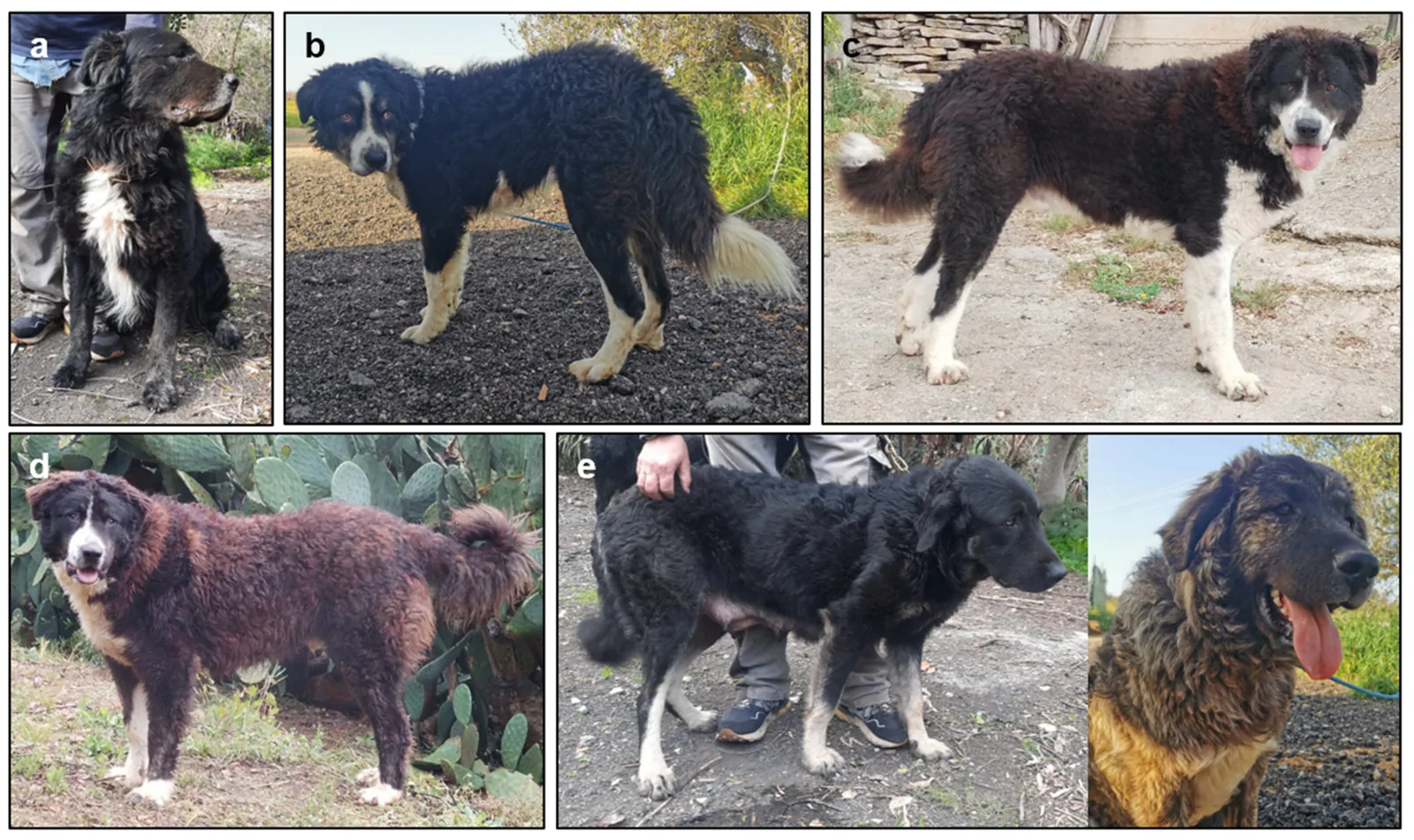
Particular coat colour features of the Sicilian Mastiff. (**a**) Brindle black and tan, with white markings and a black mask. The melanistic mask completely covers the tan points on the muzzle and head of this dog, although brindled tan points remain clearly visible on the limbs. The white marking on the chest partially hides the tan points in that area. (**b**) Brindle black and tan and white. Extensive white markings often cover the tan points on the limbs, chest, and part of the muzzle; however, brindled tan points remain visible above the eyes and partially on the muzzle. (**c**) Brindle black and tan and white with a black mask. In this dog, the brindled tan points are barely visible on the muzzle, being partially obscured by the black mask and white markings. (**d**) Example of “bronzing”, i.e., UV-induced photo-oxidation and bleaching of eumelanin pigments in the hair shaft, visible as brownish-red shades in the otherwise black areas of the back of this Brindle black and tan and white dog with a black mask. Brindled tan points remain visible on the limbs and, partially, on the muzzle and eyebrows. (**e**) The melanistic mask is usually readily identifiable on the muzzle of sable or brindle sable dogs, especially in lighter-coloured individuals (**right**), but it can also be observed in dogs with tan points, where it partially or completely covers the pheomelanic areas of the head (**left**). Conversely, the mask cannot be identified in solid eumelanic dogs.

Importantly, these discrepancies should not be interpreted as inaccuracies on the part of owners or breeders. Coat colour is generally recorded at a young age, when several traits may not yet be fully expressed or easily recognizable (Figure 3). In addition, the characteristic curly coat of the Sicilian Mastiff, seasonal shedding, environmental exposure, dirt accumulation, sun-induced bleaching (commonly called “bronzing”, Figure 4d), and age-related changes in coat appearance may all influence colour perception. Collectively, these factors make colour classification particularly challenging and emphasize the need for standardized recording guidelines.

The patterns most frequently overlooked were those involving secondary pigment distribution rather than primary coat colour. Brindling, ticking/roaning, melanistic masks, limited white markings, and, to a lesser extent, tan points were commonly omitted from colour descriptions. Among these, brindled tan points deserve particular attention. Although rarely recorded correctly, photographic evaluation suggests that Brindle black and tan represents one of the most common colour patterns in the breed. The underreporting of this phenotype is unsurprising, as brindling often obscures the underlying tan-point pattern and may become even more difficult to recognize when combined with extensive white markings or a melanistic mask (Figure 4a–c). Consequently, many dogs presenting brindled tan points appear to have been registered simply according to their predominant eumelanin colour (e.g., “Black and white”). Brindle sable represented another frequently misclassified phenotype. Unlike brindled tan points, which are often obscured by dark eumelanin pigmentation, brindle sable may vary markedly in appearance depending on age, coat length, seasonal changes, and the relative proportion of pheomelanin and eumelanin (Figure 2l,m). As a consequence, these dogs were variously recorded as Sable, Black, Brown, Gray, or Brindle, illustrating the difficulty of assigning a single colour designation to a highly variable phenotype.

The frequent use of the term “gray” deserves special consideration. This designation is explicitly mentioned in the breed standard as an accepted, although undesirable, colour [40]. Nevertheless, photographic assessment revealed that the term was applied to several genetically distinct phenotypes, including blue dilution, shaded sable, brindle sable, ticked or roan, and brown-based dogs. Descriptions such as “Black and gray” were particularly difficult to reconcile with established canine colour genetics and most likely reflect subjective visual interpretation rather than discrete phenotypic categories. Interestingly, when the breed standard was developed, gray coats were considered undesirable by the experts involved, possibly because of a perceived association with health concerns. Such perceptions may originate from the known association between certain dilution alleles and colour dilution alopecia in some breeds [50,52,53,54], although no evidence currently supports a similar relationship in the Sicilian Mastiff. Further investigation would therefore be required to clarify both the phenotypic and genetic basis of the colour category currently referred to as gray.

The identification of two smooth-coated dogs and several predominantly white individuals further illustrates the value of photographic evaluation. These traits represent disqualifying or severely undesirable features according to the breed standard [40], yet could not be reliably identified from colour records alone. Also, their frequencies may be underestimated because some dogs displaying non-permitted phenotypes may not have been entered in the registry. Although exclusion from registration based solely on coat colour is not common practice in this breed, this possibility cannot be ruled out and may be more relevant for disqualifying coat textures.

From a breed-management perspective, the discrepancy between recorded and actual phenotypes has important implications [4,44]. Population-level estimates derived exclusively from pedigree colour records may substantially underestimate the frequency of certain traits, particularly brindling, ticking, roaning, melanistic masks, and extensive white spotting. This may complicate both breeding decisions and efforts to monitor conformity with the breed standard. Standardized colour terminology, together with photographic documentation and, where appropriate, genetic testing, would greatly improve the reliability of phenotypic records and facilitate future studies of coat-colour inheritance, breed history, and population diversity.

Despite the limitations of the recorded colour descriptions, the genetic results were largely consistent with the phenotypic diversity observed within the breed. Notably, all pheomelanic individuals were attributable to the *A^y^* allele rather than recessive red; however, the relatively high frequency of the *e*^1^ allele suggests that the recessive red phenotype may occur within the wider population, although a larger sample would be needed to estimate its prevalence more accurately. Comparison with other breeds indicates that fixation of the recessive *e* allele is predominantly observed in breeds with highly restricted coat colours, including both white-coated breeds from different functional groups (e.g., Abruzzes and Maremma Shepherd Dog, West Highland White Terrier, and Bichon-type breeds) and breeds in which only uniformly pheomelanic coats are accepted or strongly predominant (e.g., some Mediterranean primitive breeds, Golden Retriever, and Vizsla). In contrast, in breeds in which multiple coat colours and/or more complex colour patterns are accepted, as is the case for the Sicilian Mastiff, pheomelanic coats are more commonly determined by the dominant *A^y^* allele rather than by recessive red, together with a broader allelic repertoire at other coat-colour loci [37].

The predominance of the *a^t^* allele was equally noteworthy. The *a^t^a^t^* genotype represented by far the most common *ASIP* genotype in the population, accounting for approximately three-quarters of tested dogs. This finding is consistent with the high frequency of tan pointed dogs identified through photographic assessment.

A high frequency of *E^m^* was also observed. While melanistic masks were identified in several dogs, they were never reported on pedigree records. In some cases, the mask may have been difficult to recognize because of dark coat colour, brindling, or extensive white markings. A small number of genotype-phenotype discrepancies were also observed, suggesting that expression of the trait may not always be completely penetrant or easily detectable. However, puppyhood photographs from the most evident discrepant cases clearly demonstrated that a melanistic mask had been present early in life and had progressively faded with age, becoming either only faintly detectable or no longer readily identifiable at the time of phenotypic assessment (Figure 3a–c). This finding further highlights the importance of evaluating coat colour across different life stages when investigating genotype–phenotype associations.

Variation in pheomelanin intensity appeared limited overall, in agreement with both the breed standard and the genetic results. The standard [40] explicitly favours light tan markings, and most Black and tan dogs displayed pale cream to fawn points. Similarly, only a small number of Sable dogs exhibited intense pheomelanin pigmentation, and notably all belonged to the same family lineage and were bred by the same person. The high frequency of the *i* allele at the *MFSD12* locus and the absence of the *II* genotype are consistent with a general tendency toward reduced pheomelanin intensity within the breed.

The occurrence of blue and brown pigmentation was supported by the presence of variants determining eumelanin dilution, whereas no evidence was found for merle, harlequin, cocoa, or albinism-associated alleles. Particularly noteworthy was the absence of Isabella dogs despite the presence of both *TYRP1* and *MLPH* recessive alleles within the population. Although this may partly reflect the limited sample size, the observation is also consistent with the fact that this colour is not included in the breed standard and may therefore have been historically selected against.

Interpretation of the genetic results should nevertheless consider several limitations of the testing platform. Most importantly, the commercial panel did not distinguish between the *K^B^* and *k^br^* alleles at the K locus, limiting our ability to assess genotype–phenotype concordance in the tested dogs. This distinction is particularly relevant in the Sicilian Mastiff because solid eumelanic and brindled phenotypes, particularly Black and white vs. Brindle black and tan and white, were among the most frequently confused colour categories. Breeders could therefore benefit from a test capable of distinguishing *K*^B^- dogs from those with the *k*^br^*k*^br^ or *k*^br^*k*^y^ genotypes.

Likewise, only a single white-spotting-associated variant was evaluated, but the complexity of white pattern inheritance in dogs can lead to the expression of variable phenotypes [23]. Consistent with this, dogs carrying one or two white-spotting-associated alleles displayed considerable phenotypic variation, ranging from minimal white markings to patterns approaching the Irish-like spotting phenotype. No dogs homozygous for the white-spotting-associated allele were identified, and no predominantly white individuals were observed among the genotyped animals. Nevertheless, the relatively high frequency of the *s^p^* allele suggests that homozygous individuals may occur within the breed and could give rise to more extensive white spotting. It should be noted that, historically, predominantly white dogs were excluded from the breed standard to distinguish the Sicilian Mastiff from the Abruzzes and Maremma Shepherd Dog, another Italian livestock guardian breed traditionally employed for similar purposes. However, current knowledge of coat colour genetics indicates that the characteristic white coat of the Abruzzes and Maremma Shepherd Dog has a different genetic basis, reflecting an extremely diluted pheomelanic coat primarily associated with recessive red at the E locus together with loci affecting pheomelanin intensity, rather than extensive white spotting associated with the S locus [15,22,37]. These observations may inform future discussions among breeders and breed associations regarding whether predominantly white coats should remain a severe fault under the breed standard, with due consideration also given to historical, functional, welfare-related, and breed-identity factors.

Different considerations apply to coat texture, as the limited occurrence of smooth-coated dogs in the population is consistent with the characteristic curly coat being effectively maintained through selection. Specifically, all tested dogs displayed curly or at least wavy coats, consistent with breed expectations. Genetic analyses on a small number of dogs revealed segregation of both the *C1* and *C2 KRT71* curl-associated variants, indicating that multiple alleles contribute to the characteristic coat texture of the breed. Consequently, testing for both currently known *KRT71* variants may provide a more complete genetic characterization and prove valuable for breeding management. The fact that the initial commercial panel screened only for *C1* highlights another area where current testing strategies could be improved. At the same time, some dogs were heterozygous for curl-associated variants. This observation, despite being based on a small sample, has practical implications for breeding, as matings between *c*-carrier animals could produce smooth-coated offspring, a disqualifying fault according to the breed standard [40]. However, it should be considered that additional, as yet unidentified, variants may also contribute to curl expression in the breed. Interestingly, the *C2 KRT71* variant has been proposed as a candidate risk factor for alopecic disorders and follicular dysplasia in other breeds [34,35]. At present, no association between this allele and follicular disorders has been reported in the Sicilian Mastiff. However, because both this variant and the dilution-associated *MLPH* variant were identified in the Sicilian Mastiff, the systematic recognition and genetic investigation of alopecic cases could help determine whether these variants have similar clinical implications in the breed. This topic may therefore be of interest for future research in the Sicilian Mastiff.

## 5. Conclusions

The present study provides the first comprehensive characterization of coat-colour and coat-texture variation in the Sicilian Mastiff by integrating pedigree records, photographic evaluation, and genetic analyses.

Our results show that registry coat colour descriptions frequently simplify complex phenotypes into a limited number of broader colour categories, leading to a substantial underestimation of the true diversity of coat colours and patterns within the breed. This may also explain the occurrence of puppies with coat colours or patterns that appear unexpected to breeders based on the registered phenotypes of their parents. These findings indicate that the coat colour registration process could be improved by integrating standardized phenotypic terminology together with the possibility for qualified experts (i.e., ENCI show judges) to verify and, when justified, update breeder-declared records. This possibility may be particularly relevant for traits that can change during growth, as observed in cases of fading eumelanic masks in the present study, and is already provided for other phenotypic traits, such as height in Dachshunds and Poodles [55,56]. Finally, the reliability of coat colour assessment could be improved by requiring registration applications, such as those for founder animals undergoing breed recognition, to include photographs taken according to standardized guidelines whenever possible.

Genetic analyses further clarified the distribution of coat-colour and coat-texture alleles within the tested dogs, while highlighting both the value and the current limitations of commercially available genetic tests. Beyond improving our understanding of pigmentation and coat-texture genetics, the estimation of allele frequencies at the breed-population level can provide scientific evidence to support the definition and periodic revision of breed standards, offering objective evidence to confirm or reconsider existing decisions [37]. This approach is also consistent with the current FCI standard format [57], which encourages the description of coat colour on a genetic basis, thereby promoting standards that more accurately reflect the underlying genetic architecture of the breed.

Overall, the combined implementation of the approaches proposed in this study would increase the accuracy and consistency of phenotypic records, support more informed breeding decisions, and facilitate future studies on coat colour inheritance, breed history, and genetic diversity.

## Figures and Tables

**Table 1:** Frequency of dogs heterozygous or homozygous for the tested alleles related to coat colour and characteristics.

Trait	Gene	Variant	% Heterozygous	% Homozygous	Phenotype
Extension	*MC1R*	*E^m^*	32.65% (16/49)	10.20% (5/49)	Melanistic mask
		*E^G^*	0%	0%	Grizzle/domino
		*e^A^*	6.12% (3/49)	0%	Ancient red (variable coat, [17])
		*e^H^*	2.04% (1/49)	0%	Recessive red
		*e* ^1^	18.37% (9/49)	0%	Recessive red
		*e* ^2^	0%	0%	Recessive red
		*e* ^3^	0%	0%	Recessive red
Agouti	*ASIP* *	*A^y^*	25.53% (12/47)	0%	Dominant yellow and shaded yellow
		*a^t^*	25.53% (12/47)	74.46 (35/47)	Black back (tan points)
		*a*	0%	0%	Recessive black
Kurokami	*CBD103* ^†^	*K^B^* or *k^br^*	74.00% (37/50)	0%	Dominant black or Brindle
Dilution	*MLPH*	*d* ^1^	51.02% (25/49)	12.24% (6/49)	Diluted eumelanin (black to blue)
		*d* ^2^	0%	0%	Diluted eumelanin (black to blue)
		*d* ^3^	0%	0%	Diluted eumelanin (black to blue)
Intensity	*MFSD12*	*i*	24.00% (12/50)	76.00% (38/50)	Light pheomelanin (cream, white)
Brown	*TYRP1*	*b^c^*	2.04% (1/49)	0%	Diluted eumelanin (black to brown)
		*b^s^*	34.69% (17/49)	4.08% (2/49)	Diluted eumelanin (black to brown)
		*b^d^*	0%	0%	Diluted eumelanin (black to brown)
		*b^asd^*	0%	0%	Diluted eumelanin (black to brown)
		*b^e^*	0%	0%	Diluted eumelanin (black to brown)
		*b^h^*	0%	0%	Diluted eumelanin (black to brown)
Cocoa	*HSP3*	co	0%	0%	Diluted eumelanin (black to cocoa)
White spotting	*MITF* ^‡^	*s^p^*	38.00% (19/50)	0%	White spotting (piebald)
Roan/Ticking	*USH2A*	*T^r^*	8.00% (4/50)	0%	Roan (linkage test)
Merle	*PMEL*	M	0%	0%	Merle
Harlequin	*PSMB7*	H	0%	-	Harlequin
Black saddle	*RALY*	WT	43.75% (21/48)	6.25% (3/48)	Possible association with saddle tan
Albinism	*SLC45A2*	*c^al^*	0%	0%	Albino
Hair length	*FGF5*	*lh* ^1^	6.12% (3/49)	93.88% (46/49)	Long hair
		*lh* ^2^	0%	0%	Long hair
		*lh* ^3^	2.04% (1/49)	0%	Long hair
		*lh* ^4^	0%	0%	Long hair
		*lh* ^5^	0%	0%	Long hair
Curly hair	*KRT71* ^§^	*C1*	36.73% (18/49)	0%	Curly hair
Furnishing	*RSPO2*	F	0%	0%	Furnishing
Hypotrichosis	*FOXI3*	*Hr^cc^*	0%	-	Hypotrichosis
Hypotrichosis	*SGK3*	*hr^aht^*	0%	0%	Hypotrichosis
		*hr^sd^*	0%	0%	Hypotrichosis
Degree of shedding	*MC5R* ^¶^	*sd*	10.42% (5/48)	2.08% (1/48)	Reduced shedding
Hair ridge	*FGF3, FGF4, FGF19, ORAOV1*	R	0%	0%	Hair ridge

Percentages were calculated separately for each variant using only dogs with a successful genotype call. The denominator shown in parentheses therefore represents the number of successfully genotyped dogs for that variant and may vary because failed or missing genotype calls were excluded. * An alternative nomenclature for the A locus indicates: *DY* and *SY* (*A^y^*) for dominant yellow and shaded yellow, respectively; *AG* (*a^w^*) for agouti; *BS* and *BB* (*a^t^*) for black saddle and black back, respectively; and *a* for recessive black [12]. However, to maintain consistency with the nomenclature used in the genetic test, the classical nomenclature was retained throughout this study. Genotypes were not reported for three individuals because they appeared to carry two *a^t^* alleles and one *a* allele, most likely due to a laboratory error. However, this does not affect the corresponding phenotype classification, as the presence of *a^t^* determines expression of the *a^t^* phenotype. ^†^ The test cannot distinguish between *K^B^* and *k^br^* alleles. ^‡^ Only the *MITF*-associated white spotting variant (historically referred to as *s^p^*) was tested. Historically, white spotting was thought to be controlled by the incomplete dominant allelic series *S*, *s^i^*, *s^p^*, and *s^w^*, corresponding to no white spotting, Irish spotting, piebald spotting, and extreme white, respectively. Current evidence indicates that white spotting is determined by regulatory variation at the *MITF* locus together with additional modifier genes. Accordingly, the phenotypic effect of the tested variant varies according to breed-specific genetic background, and the same genotype may be associated with substantially different white spotting phenotypes both within and among breeds [8,23]. ^§^ MyDogDNA did not include *C2* test. Additional tests on nine subjects for both *C1* and *C2* variants identified one dog with one *C1* copy and three with one *C2* copy; two homozygous *C2C2* dogs; and three *C1C2* dogs. ^¶^ The allele tested by MyDogDNA is reported as *sd* and is associated with reduced shedding. According to the test interpretation, dogs carrying zero, one, or two copies of *sd* are classified as seasonal, occasional, or reduced shedders, respectively.

## Data Availability

The original contributions presented in this study are included in the article and Supplementary Material. Further inquiries can be directed to the corresponding authors.

## Supplementary Materials

The following supporting information can be downloaded at: https://www.mdpi.com/article/10.3390/vetsci13090958/s1.

## References

[1] Lindblad-Toh K., Wade C.M., Mikkelsen T.S., Karlsson E.K., Jaffe D.B., Kamal M., Clamp M., Chang J.L., Kulbokas E.J., Zody M.C. (2005). Genome sequence, comparative analysis and haplotype structure of the domestic dog. Nature.

[2] Ash E.C. (1927). Dogs: Their History and Development.

[3] Ostrander E.A. (2007). Genetics and the Shape of Dogs. Am. Sci..

[4] Brancalion L., Haase B., Wade C.M. (2022). Canine coat pigmentation genetics: A review. Anim. Genet..

[5] Derry M. (2018). White Collies, Beauty or Genetic Defect: Regulation and Breeding, 1870–2013. Soc. Anim..

[6] Cadieu E., Neff M.W., Quignon P., Walsh K., Chase K., Parker H.G., VonHoldt B.M., Rhue A., Boyko A., Byers A. (2009). Coat Variation in the Domestic Dog Is Governed by Variants in Three Genes. Science.

[7] Schmutz S.M., Berryere T.G. (2007). Genes affecting coat colour and pattern in domestic dogs: A review. Anim. Genet..

[8] Karlsson E.K., Baranowska I., Wade C.M., Salmon Hillbertz N.H., Zody M.C., Anderson N., Biagi T.M., Patterson N., Pielberg G.R., Kulbokas E.J. (2007). Efficient Mapping of Mendelian Traits in Dogs Through Genome-Wide Association. Nat. Genet..

[9] Slominski A., Zmijewski M.A., Pawelek J. (2012). L-tyrosine and L-dihydroxyphenylalanine as hormone-like regulators of melanocyte functions. Pigment Cell Melanoma Res..

[10] Newton J.M., Wilkie A.L., He L., Jordan S.A., Metallinos D.L., Holmes N.G., Jackson I.J., Barsh G.S. (2000). Melanocortin 1 receptor variation in the domestic dog. Mamm. Genome.

[11] Belyakin S., Maksimov D.A., Pobedintseva M.A., Laktionov P.P., Voronova D. (2022). ASIP promoter variants predict sesame coat color in Shiba Inu dogs. Vet. Sci..

[12] Bannasch D.L., Kaelin C.B., Letko A., Loechel R., Hug P., Jagannathan V., Henkel J., Roosje P., Hytönen M.K., Lohi H. (2021). Dog colour patterns explained by modular promoters of ancient canid origin. Nat. Ecol. Evol..

[13] Leonard B.C., Marks S.L., Outerbridge C.A., Affolter V.K., Kananurak A., Young A., Moore P.F., Bannasch D.L., Bevins C.L. (2012). Activity, expression and genetic variation of canine β-defensin 103: A multifunctional antimicrobial peptide in the skin of domestic dogs. J. Innate Immun..

[14] Candille S.I., Kaelin C.B., Cattanach B.M., Yu B., Thompson D.A., Nix M.A., Kerns J.A., Schmutz S.M., Millhauser G.L., Barsh G.S. (2007). A β-defensin mutation causes black coat color in domestic dogs. Science.

[15] Dürig N., Letko A., Lepori V., Hadji Rasouliha S., Loechel R., Kehl A., Hytönen M.K., Lohi H., Mauri N., Dietrich J. (2018). Two MC1R loss-of-function alleles in cream-coloured Australian Cattle Dogs and white Huskies. Anim. Genet..

[16] Dreger D.L., Schmutz S.M. (2010). A new mutation in MC1R explains a coat color phenotype in 2 “old” breeds: Saluki and Afghan Hound. J. Hered..

[17] Anderson H., Honkanen L., Ruotanen P., Mathlin J., Donner J. (2020). Comprehensive genetic testing combined with citizen science reveals a recently characterized ancient MC1R mutation associated with partial recessive red phenotypes in dog. Canine Med. Genet..

[18] Schmutz S.M., Berryere T.G., Goldfinch A.D. (2002). TYRP1 and MC1R genotypes and their effects on coat color in dogs. Mamm. Genome.

[19] Bauer A., Kehl A., Jagannathan V., Leeb T. (2018). A novel MLPH variant in dogs with coat colour dilution. Anim. Genet..

[20] Philipp U., Hamann H., Mecklenburg L., Nishino S., Mignot E., Günzel-Apel A.R., Schmutz S.M., Leeb T. (2005). Polymorphisms within the canine MLPH gene are associated with dilute coat color in dogs. BMC Genet..

[21] Weich K., Affolter V., York D., Rebhun R., Grahn R., Kallenberg A., Bannasch D. (2020). Pigment intensity in dogs is associated with a copy number variant upstream of KITLG. Genes.

[22] Hédan B., Cadieu E., Botherel N., de Citres C.D., Letko A., Rimbault M., Drögemüller C., Jagannathan V., Derrien T., Schmutz S. (2019). Identification of a missense variant in MFSD12 involved in dilution of phaeomelanin leading to white or cream coat color in dogs. Genes.

[23] Baranowska Körberg I., Sundström E., Meadows J.R.S., Rosengren Pielberg G., Gustafson U., Hedhammar Å., Karlsson E.K., Seddon J., Söderberg A., Vilà C. (2014). A Simple Repeat Polymorphism in the MITF-M Promoter Is a Key Regulator of White Spotting in Dogs. PLoS ONE.

[24] Brancalion L., Haase B., Mazrier H., Willet C.E., Lindblad-Toh K., Lingaas F., Wade C.M. (2021). Roan, ticked and clear coat patterns in the canine are associated with three haplotypes near usherin on CFA38. Anim. Genet..

[25] Kawakami T., Jensen M.K., Slavney A., Deane P.E., Milano A., Raghavan V., Ford B., Chu E.T., Sams A.J., Boyko A.R. (2021). R-locus for roaned coat is associated with a tandem duplication in an intronic region of USH2A in dogs and also contributes to Dalmatian spotting. PLoS ONE.

[26] Varga L., Lénárt X., Zenke P., Orbán L., Hudák P., Ninausz N., Pelles Z., Szőke A. (2020). Being merle: The molecular genetic background of the canine merle mutation. Genes.

[27] Ballif B.C., Emerson L.J., Ramirez C.J., Carl C.R., Sundin K., Flores-Smith H., Shaffer L.G. (2021). The PMEL gene and merle (dapple) in the dachshund: Cryptic, hidden, and mosaic variants demonstrate the need for genetic testing prior to breeding. Hum. Genet..

[28] Clark L.A., Tsai K.L., Starr A.N., Nowend K.L., Murphy K.E. (2011). A missense mutation in the 20S proteasome β2 subunit of Great Danes having harlequin coat patterning. Genomics.

[29] Wijesena H.R., Schmutz S.M. (2015). A missense mutation in SLC45A2 is associated with albinism in several small long haired dog breeds. J. Hered..

[30] Caduff M., Bauer A., Jagannathan V., Leeb T. (2017). OCA2 splice site variant in German Spitz dogs with oculocutaneous albinism. PLoS ONE.

[31] Bychkova E., Viktorovskaya O., Filippova E., Eliseeva Z., Barabanova L., Sotskaya M., Markov A. (2021). Identification of a candidate genetic variant for the Himalayan color pattern in dogs. Gene.

[32] Parker H.G., Chase K., Cadieu E., Lark K.G., Ostrander E.A. (2010). An insertion in the RSPO2 gene correlates with improper coat in the Portuguese water dog. J. Hered..

[33] Housley D.J.E., Venta P.J. (2006). The long and the short of it: Evidence that FGF5 is a major determinant of canine ’hair’-itability. Anim. Genet..

[34] Salmela E., Niskanen J., Arumilli M., Donner J., Lohi H., Hytönen M.K. (2019). A novel KRT71 variant in curly-coated dogs. Anim. Genet..

[35] Bauer A., Hadji Rasouliha S., Brunner M.T., Jagannathan V., Bucher I., Bannoehr J., Varjonen K., Bond R., Bergvall K., Welle M.M. (2019). A second KRT71 allele in curly coated dogs. Anim. Genet..

[36] Arman K. (2007). A new direction for kennel club regulations and breed standards. Can. Vet. J..

[37] Dreger D.L., Hooser B.N., Hughes A.M., Ganesan B., Donner J., Anderson H., Holtvoigt L., Ekenstedt K.J. (2019). True Colors: Commercially-acquired morphological genotypes reveal hidden allele variation among dog breeds, informing both trait ancestry and breed potential. PLoS ONE.

[38] Conant E.K., Juras R., Cothran E.G. (2011). Incidence of the mask phenotype M264V mutation in Labrador Retrievers. Res. Vet. Sci..

[39] Liotta L., Bionda A., Cortellari M., Negro A., Crepaldi P. (2021). From phenotypical to genomic characterisation of the mannara dog: An italian shepherd canine resource. Ital. J. Anim. Sci..

[40] ENCI Standard di razza del Mastino Siciliano-Cane di Mannara 2022. http://www.enci.it/media/7591/903.pdf.

[41] Kang D., Park W., Kim M., Lim Y.J., Kim J.S., Oh S., Plassais J., Kim J., Choi B.H. (2025). Deep sequencing of Korean Jindo dog reveals evolutionary trajectory of coat color variations. Genomics.

[42] Cortellari M., Bionda A., Talenti A., Ceccobelli S., Attard G., Lasagna E., Crepaldi P., Liotta L. (2021). Genomic variability of Cirneco dell’Etna and the genetic distance with other dog breeds. Ital. J. Anim. Sci..

[43] Wiener P., Sánchez-Molano E., Clements D.N., Woolliams J.A., Haskell M.J., Blott S.C. (2017). Genomic data illuminates demography, genetic structure and selection of a popular dog breed. BMC Genom..

[44] Marín Navas C., Navas González F.J., Castillo López V., Payeras Capellà L., Gómez Fernández M., Delgado Bermejo J.V. (2020). Impact of breeding for coat and spotting patterns on the population structure and genetic diversity of an islander endangered dog breed. Res. Vet. Sci..

[45] Björnerfeldt S., Hailer F., Nord M., Vilà C. (2008). Assortative mating and fragmentation within dog breeds. BMC Evol. Biol..

[46] Ducrest A.L., Keller L., Roulin A. (2008). Pleiotropy in the melanocortin system, coloration and behavioural syndromes. Trends Ecol. Evol..

[47] Amat M., Manteca X., Mariotti V.M., Ruiz de la Torre J.L., Fatjó J. (2009). Aggressive behavior in the English cocker spaniel. J. Vet. Behav..

[48] van Rooy D., Wade C.M. (2019). Association between coat colour and the behaviour of Australian Labrador retrievers. Canine Genet. Epidemiol..

[49] Kim Y.K., Lee S.S., Oh S.I., Kim J.S., Suh E.H., Houpt K.A., Lee H.C., Lee H.J., Yeon S.C. (2010). Behavioural reactivity of the Korean native Jindo dog varies with coat colour. Behav. Process..

[50] Caramalac S.M., Caramalac S.M., Babo-Terra V.J., Ramos C.A.N., Palumbo M.I.P. (2021). PCR-RFLP molecular confirmation of color dilution alopecia in dogs in Brazil. J. Vet. Diagn. Investig..

[51] Karyadi D.M., Karlins E., Decker B., vonHoldt B.M., Carpintero-Ramirez G., Parker H.G., Wayne R.K., Ostrander E.A. (2013). A copy number variant at the KITLG locus likely confers risk for canine squamous cell carcinoma of the digit. PLoS Genet..

[52] Kim J.H., Kang K., Sohn H.J., Woo G.H., Jean Y.H., Hwang E.K. (2005). Color-dilution alopecia in dogs. J. Vet. Sci..

[53] Beco L., Fontaine J., Gross T.L., Charlier G. (1996). Colour dilution alopecia in seven Dachshunds. A clinical study and the hereditary, microscopical and ultrastructural aspect of the disease. Vet. Dermatol..

[54] Miller W.H. (1990). Colour Dilution Alopecia in Doberman Pinschers with Blue or Fawn Coat Colours: A Study on the Incidence and Histopathology of this Disorder. Vet. Dermatol..

[55] ENCI Regolamento conferma di taglia razza Barbone 2024. https://www.enci.it/media/9180/regolamento-per-la-conferma-di-taglia-razza-barbone.pdf.

[56] ENCI Regolamento per la conferma di taglia del Bassotto tedesco 2023. https://www.enci.it/media/6137/regolamento-per-la-conferma-di-taglia-del-bassotto-tedesco.pdf.

[57] FCI Internal Rules of the FCI-Enclosure 6–FCI Model Standard 2023. https://www.fci.be/medias/FCI-REG-ROI-STA-MOD-ANN-006-en-15832.docx.

